# Hepatectomy versus transcatheter arterial chemoembolization for resectable BCLC stage A/B hepatocellular carcinoma beyond Milan criteria: A randomized clinical trial

**DOI:** 10.3389/fonc.2023.1101162

**Published:** 2023-02-27

**Authors:** Chongkai Fang, Rui Luo, Ying Zhang, Jinan Wang, Kunliang Feng, Silin Liu, Chuyao Chen, Ruiwei Yao, Hanqian Shi, Chong Zhong

**Affiliations:** ^1^ The First Clinical Medical School, Guangzhou University of Chinese Medicine, Guangzhou, China; ^2^ The First Affiliated Hospital, Guangzhou University of Chinese Medicine, Guangzhou, China; ^3^ Lingnan Medical Research Center of Guangzhou University of Chinese Medicine, Guangzhou, China

**Keywords:** hepatocellular carcinoma, neoadjuvant treatment, transcatheter arterial chemoembolization, hepatectomy, survival, Milan criteria

## Abstract

**Background:**

Hepatectomy is the recommended option for radical treatment of BCLC stage A/B hepatocellular carcinoma (HCC) that has progressed beyond the Milan criteria. This study evaluated the efficacy and safety of preoperative neoadjuvant transcatheter arterial chemoembolization (TACE) for these patients.

**Methods:**

In this prospective, randomized, open-label clinical study, BCLC stage A/B HCC patients beyond the Milan criteria were randomly assigned (1:1) to receive either neoadjuvant TACE prior to hepatectomy (NT group) or hepatectomy alone (OP group). The primary outcome was overall survival (OS), while the secondary outcomes were progression-free survival (PFS) and adverse events (AEs).

**Results:**

Of 249 patients screened, 164 meeting the inclusion criteria were randomly assigned to either the NT group (n = 82) or OP group (n = 82) and completed follow-up requirements. Overall survival was significantly greater in the NT group compared to the OP group at 1 year (97.2% vs. 82.4%), two years (88.4% vs. 60.4%), and three years (71.6% vs. 45.7%) (p = 0.0011) post-treatment. Similarly, PFS was significantly longer in the NT group than the OP group at 1 year (60.1% vs. 39.9%), 2 years (53.4% vs. 24.5%), and 3 years (42.2% vs. 24.5%) (p = 0.0003). No patients reported adverse events of grade 3 or above in either group.

**Conclusions:**

Neoadjuvant TACE prolongs the survival of BCLC stage A/B HCC patients beyond the Milan criteria without increasing severe adverse events frequency.

**Clinical trial registration:**

https://www.chictr.org.cn/, identifier ChiCTR2200055618.

## Introduction

Hepatocellular carcinoma (HCC) is the sixth most prevalent cancer and the third most common cause of cancer-related death worldwide (1, 2). Current treatments for HCC include surgical resection, liver transplantation, and local radiofrequency ablation, of which hepatectomy is the most frequently used method for radical treatment. However, the recurrence rate is approximately 50%–60% at 2 years and 80% at 5 years, and the median survival time after recurrence without additional therapeutic interventions is only 2.7 to 4.0 months (3–7). Therefore, it is necessary to explore additional treatments to improve survival among this patient group. Barcelona Clinic Liver Cancer (BCLC) staging is a common clinical standard for assessing clinical progression and treatment selection according to tumor size, tumor number, degree of liver function, and general physical condition (8). The recommended treatments for very early and early BCLC stages (0 and A) include surgical resection, local radiofrequency ablation, and liver transplantation, while the recommended treatments for intermediate stage (B) included transcatheter arterial chemoembolization (TACE) (9). The Milan criteria are widely used for evaluating eligibility for liver transplantation to treat early-stage liver cancer transplantation, but most patients are beyond Milan criteria by the time of diagnosis (10, 11). Moreover, some patients meeting the Milan criteria miss the opportunity for liver transplantation while waiting for a suitable donor. Also, liver cancer progresses quickly, so changes in size and location can increase the risks of surgery. Therefore, for patients with stage A/B liver cancer beyond Milanese standard BCLC, we use neoadjuvant TACE to control tumor progression before surgical resection. In our preliminary clinical observation, this treatment not only reduced the risks of surgery but also improved overall survival (OS).

Nevertheless, preoperative TACE remains controversial for resectable HCC. Some investigators have reported that preoperative TACE increases the risk of tumor cells metastasizing into the bloodstream without improving the survival in patients with resectable solitary HCC (12). In contrast, others have reported that preoperative TACE combined with hepatectomy improves both OS and progression-free survival (PFS) of patients with giant HCC compared to hepatectomy alone (13). Therefore, it is essential to evaluate if preoperative TACE can benefit patients with BCLC stage A/B HCC who are beyond Milan criteria.

This prospective clinical trial of patients diagnosed with BCLC stage A/B HCC beyond Milan criteria was designed to evaluate the clinical safety and efficacy of TACE combined with hepatectomy prior to hepatectomy alone.

## Methods

### Trial design

This open-label, phase III, randomized, parallel study was conducted at the First Affiliated Hospital of Guangzhou University of Chinese Medicine (Guangzhou, China) and Sun Yat-Sen Cancer Center (Guangzhou, China). Patients with HCC beyond Milan criteria were randomized to receive neoadjuvant TACE plus hepatectomy (NT group) or hepatectomy alone (OP group). The study was conducted in accordance with the Declaration of Helsinki and CSCO Clinical Practice Guidelines and was approved by the First Affiliated Hospital of Guangzhou University of Chinese Medicine Institutional Review Board and Ethics Committee (IRB approval number: NO.ZYYECK [2019]163). All patients participated voluntarily and provided informed written consent. Patients meeting the eligibility criteria (below) were randomized at a 1:1 ratio using a sealed envelope system. An application for registration was submitted to the Chinese Clinical Trial Registry (https://www.chictr.org.cn/, trail number: ChiCTR2200055618).

### Eligibility criteria

Inclusion criteria were as follows: (1) 18–75 years of age; (2) Eastern Cooperative Oncology Group performance score (ECOG PS) of 0 or 1; (3) BCLC A/B stage exceeding the Milan criteria; (4) HCC lesion(s) not previously treated with local or systematic therapy; (5) meeting criteria for Child Pugh class A live score; (6) no distant metastasis, organ dysfunction, or other contraindications to liver resection; (7) laboratory tests meeting the acceptance criteria for TACE and liver resection; (8) no allergy to any TACE agent; (9) informed written consent; and (10) no concomitant antitumor therapy or enrollment in other clinical trials.

The exclusion criteria were as follows: (1) mixed tumors exhibiting other features; (2) recurrent HCC or other simultaneously occurring malignancies; (3) received alternatives to TACE or palliative resection for anticancer treatment before hepatectomy; (4) serious major organ dysfunction; (5) lack of clinical and follow-up data; and (6) death from unrelated causes.

### Neoadjuvant TACE

Patients in the NT group received at least two times TACE before hepatectomy. Hepatic angiography was performed by inserting a catheter through the femoral artery using the Seldinger technique. After assessing the hepatic vascular anatomy, TACE was performed selectively through the left or right hepatic artery, or the tumor-feeding artery when technically possible (as there was a need for super selective catheterization in some cases). Epirubicin (30 mg/m^2^) and lipiodol (5–20 ml) emulsions were injected into the tumor, with a lipiodol dose set according to tumor diameter. The manufacturer of chemotherapeutic agents allowed diverse selection due to TACE was performed at different hospitals. Approximately 4–6 weeks after the initial therapy, a complete assessment was conducted consisting of a physical examination, routine blood analysis, and computed tomography (CT) scan. Based on this review and patient condition, the decision was made to perform the second cycle of TACE.

After neoadjuvant therapy, we estimated the efficiency of TACE by radiography based on the Modified Response Evaluation Criteria In Solid Tumors (mRECIST). If the patient was diagnosed with progressive disease (PD) or could not accept the hepatectomy, we would suggest the appropriate advice for the subsequent therapy. On the contrary, if the patient achieved complete remission (CR), partial remission (PR), or stable disease (SD), resection was recommended as the first optional treatment.

### Partial hepatectomy

Anatomic resection was conducted using Pringle’s maneuver to limit liver blood volume inflow and thereby reduce uncontrolled bleeding. Briefly, an elastic tourniquet was tightened around the entire hepatoduodenal ligament with occlusive time set according to liver function (up to 30 min if the liver function was excellent).

### Follow-up

Patients were evaluated at least every 3 months during the first 2 years post-hepatectomy and every 6 months thereafter. If patients could not review their tumor condition, we actively connect with them by telephone or mail for follow-up. Ultrasonography, chest X-ray, CT, magnetic resonance imaging (MRI), serum alpha-fetoprotein (AFP) measurement, liver function tests, and blood analyses were conducted routinely as part of the standard diagnostic process. After detecting a suspected recurrence/metastasis, further tests were performed, including hepatic angiography or biopsy. Recurrence/metastasis was confirmed by cytologic/histologic evidence or noninvasive diagnostic criteria established by the European Association for the Study of Liver. All patients with recurrence were subsequently treated by our hospital’s multi-disciplinary team according to tumor location, liver function, and physical condition.

We strictly recorded every adverse event (AE) during the whole stage of treatment. AEs associated with TACE and hepatectomy were evaluated according to National Cancer Institute Common Terminology Criteria for Adverse Events v4.0.

### Statistical analyses

The primary outcome measure was OS from the day the patient was randomly assigned to a treatment group until the date of death from any cause, while the secondary outcomes were PFS and AEs.

From our retrospect research, the OS of 3 years between the NT and OP groups was approximately 66% and 40%, respectively. Following the principle that the primary outcome should get 90% statistical power and differ between intervention groups by one-sided α = 0.05, we recruited 249 patients and randomized eligible patients equally into the NT and OP groups. Assuming 10% loss to follow-up, we estimated that it would require 81 cases per group randomized by PASS [Hintze, J. (2011). PASS 11, NCSS, LLC, Kaysville, UT, USA. www.ncss.com]. Survival was plotted using the Kaplan–Meier method and compared between groups using the log-rank test, while Cox proportional hazards analysis was conducted to calculate hazard ratios (HRs) with 95% confidence intervals (CIs). Survival curves and forest plot were drawn and analyzed using GraphPad Prism version 8.0.1 for Windows (GraphPad Software San Diego, CA, USA). AEs were compared between groups by independent samples *t*-test. Subgroup analyses included sex, age, tumor size, cirrhosis, AFP, and hepatitis B or C virus (HBV or HCV) infection as potential prognostic factors. These analyses were conducted using SPSS software version 25 (Chicago, IL, USA). p < 0.05 was considered statistically significant for all tests.

## Results

### Patient characteristics and treatment

**Figure 1:**
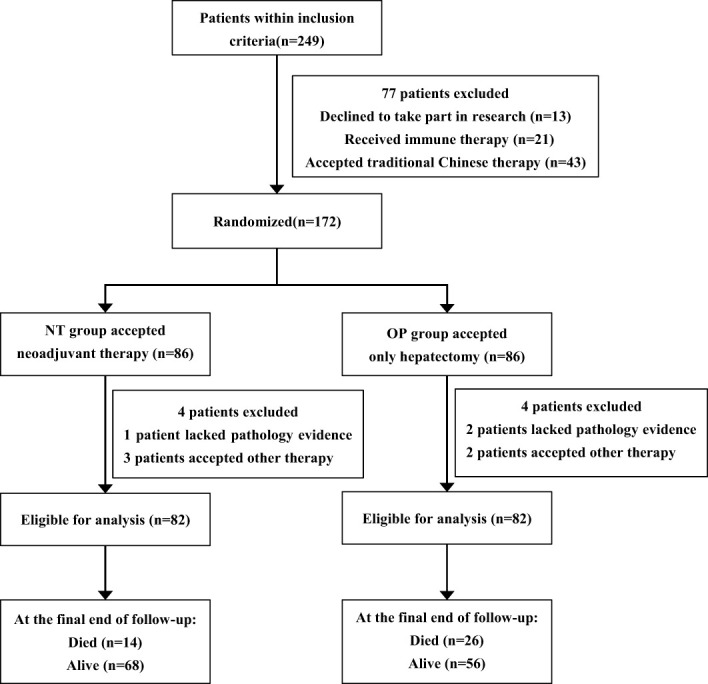
Patient enrollment and randomization to treatment groups.

Between 2 April 2020, and 2 April 2021, 249 patients were screened, and 172 (intention-to-treat population) were randomly assigned to receive neoadjuvant therapy prior to hepatectomy (n = 86) or hepatectomy alone (n = 86). In the NT group, one patient lacked pathology evidence, and three patients accepted other therapies. In the OP group, two patients lacked the pathology evidence, and two patients received other treatments. Finally, the efficacy and safety analyses included 82 patients in each group (**Figure 1**).

Baseline demographic and disease characteristics did not differ significantly between groups with the exception of higher cirrhosis incidence in the OP group (**Table 1**).

**Table 1:** Clinical characteristics of patients in the neoadjuvant group (NT group) and hepatectomy alone group (OP group).

		Total	NT group	OP group	p-Value
Patients (n)		164	82	82	-
**Gender (n%)**	**male**	141 (86.0%)	68 (82.9%)	73 (89.0%)	0.261
	**female**	23 (14.0%)	14 (17.1%)	9 (11.0%)	
**Age (n%)**	**<65**	137 (83.5%)	71 (86.6%)	66 (80.5%)	0.292
	**≥65**	27 (16.5%)	11 (13.4%)	16 (19.5%)	
**BCLC (n%)**	**BCLC A**	107 (65.2%)	53 (64.6%)	54 (65.9%)	0.87
	**BCLC B**	57 (34.8%)	29 (35.4%)	28 (34.1%)	
**Number of tumor (s)**	**Single**	106 (64.6%)	53 (64.6%)	53 (64.6%)	1
	**Multiple**	58 (35.4%)	29 (35.4%)	29 (35.4%)	
**Maximum diameter of tumor (cm)**	**≤5cm**	25 (15.2%)	12 (14.6%)	13 (15.9%)	0.828
	**>5cm**	139 (84.8%)	70 (85.4%)	69 (84.1%)	
**Hepatitis B/C infection**	**Y**	145 (88.4%)	73 (89.0%)	72 (87.8%)	0.807
	**N**	19 (11.6%)	9 (11.0%)	10 (12.2%)	
**Cirrhosis**	**Y**	59 (36.0%)	19 (23.2%)	40 (48.8%)	0.001
	**N**	105 (64.0%)	63 (76.8%)	42 (51.2%)	
**Differentiation of tumor**	**1**	6 (3.7%)	6 (7.3%)	0 (0%)	0.401
	**2**	90 (54.9%)	43 (52.4%)	47 (57.3%)	
	**3**	68 (41.5%)	33 (40.2%)	35 (42.7%)	
**Child-Pugh (n%)**	**A**	164 (100%)	82 (100%)	82 (100%)	–
	**B**	0 (0%)	0 (0%)	0 (0%)	
**AFP**	**<400**	89 (54.3%)	39 (47.6%)	50 (61.0%)	0.085
	**≥400**	75 (45.7)	43 (52.4%)	32 (39.0%)	
**Serum biomarker**	**NEU**	4.05 (2.98–5.20)	4.07 (3.02–5.53)	3.99 (2.93–4.71)	0.26
	**WBC**	6.51 (5.27–7.88)	6.30 (5.24–7.95)	6.67 (5.29–7.73)	0.784
	**HGB**	146 (133.3–156)	143 (133–152)	148 (138–159)	0.107
	**PLT**	213 (164.3–279.3)	216 (169–301)	208 (161–274)	0.436
	**ALT**	38.3 (26.7–60.7)	37.55 (25.00–59.50)	39.45 (28.92–63.10)	0.379
	**ALB**	42.5 (40.3–44.5)	41.85 (39.67–44.00)	43.15 (40.57–44.95)	0.107
	**TB**	12.7 (10.1–15.6)	13.0 (10.5–16.3)	12.3 (9.3–15.3)	0.076
	**PT**	11.8 (11.3–12.6)	12.0 (11.3–12.8)	11.6 (11.2–12.5)	0.091
	**CREA**	75.9 (65.1–85.5)	76.60 (62.35–86.25)	75.35 (66.82–85.17)	0.653

AFP, alpha-fetoprotein; NEU, neutrophil; WBC, white blood cells; HGB, hemoglobin; PLT, platelet count; ALT, alanine aminotransferase; ALB, albumin; TBil, total bilirubin; PT, prothrombin time; CREA, creatinine.

### Efficacy analysis

**Figure 2:**
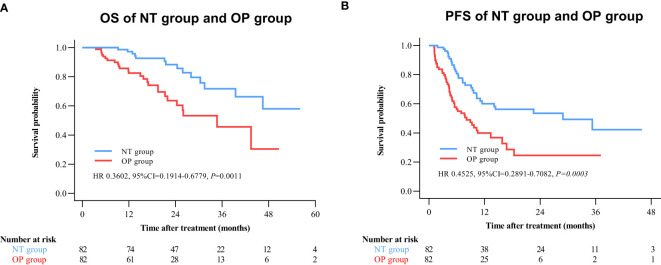
Kaplan–Meier survival curves of the neo-adjuvant plus hepatectomy (NT) group and hepatectomy alone (OP) group. **(A)** Overall survival (OS). **(B)** Progression-free survival (PFS).

**Figure 3:**
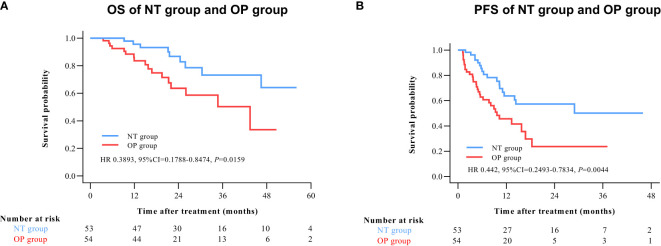
Kaplan–Meier survival curves of BCLC A patients in the NT and OP groups. **(A)** Overall survival (OS). **(B)** Progression-free survival (PFS).

**Figure 4:**
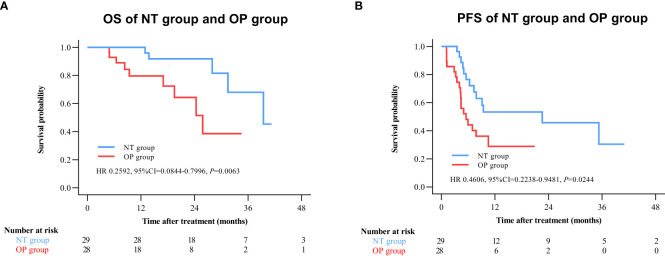
Kaplan–Meier survival curves of BCLC B patients in the NT and OP groups. **(A)** Overall survival (OS). **(B)** Progression-free survival (PFS).

By 2 April 2021, there were 14 deaths and 31 recurrences in the NP group compared to 26 deaths and 48 recurrences in the OP group. Overall survival was higher in the NT group compared to the OP at 1 year (97.2% vs. 82.4%), 2 years (88.4% vs. 60.4%), and 3 years (71.8% vs. 45.7%) post-treatment (HR 0.3602 [95% CI, 0.1914 to 0.6779]; p = 0.0011) (**Figure 2A**). Progression-free survival was also greater in the NT group compared to the OP at 1 year (60.1% vs. 39.9%), 2 years (53.4% vs. 24.5%), and 3 years (42.2% vs. 24.5%) post-treatment (HR 0.4525 [95% CI, 0.2891 to 0.7082]; p = 0.0003) (**Figure 2B**). Among patients with (earlier) BCLC A stage disease, OS was higher in the NT group than the OP group at 1 year (95.6% vs. 83.6%), 2 years (82.9% vs. 63.6%), and 3 years (73.3% vs. 50.3%) post-treatment (HR 0.3893 [95%CI, 0.1788 to 0.8474]; p = 0.0159) (**Figure 3A**). Similarly, PFS was higher among NT group patients with BCLC A stage disease compared to OP group patients with BCLC A stage disease at 1 year (63.7% vs. 45.7%), 2 years (57.4% vs. 23.7%), and 3 years (50.2% vs. 23.7%) post-treatment (HR 0.442 [95%CI, 0.2493 to 0.7834]; p = 0.0044) (**Figure 3B**). Among patients with intermediate BCLC B stage disease as well, OS was higher in the NT group at 1 year (100% vs. 97.6%), 2 years (91.8% vs. 64.3%), and 3 years (68% vs. 38.6%) post-treatment (HR 0.2592 [95%CI, 0.0844 to 0.7996]; p = 0.0063) (**Figure 4A**), as was PFS at 1 year (53.3% vs. 28.9%), 2 years (45.7% vs. 28.9%), and 3 years (30.5% vs. 28.9%) post-treatment (HR 0.4606 [95% CI, 0.2238–0.9481]; p = 0.0244) (**Figure 4B**).

**Figure 5:**
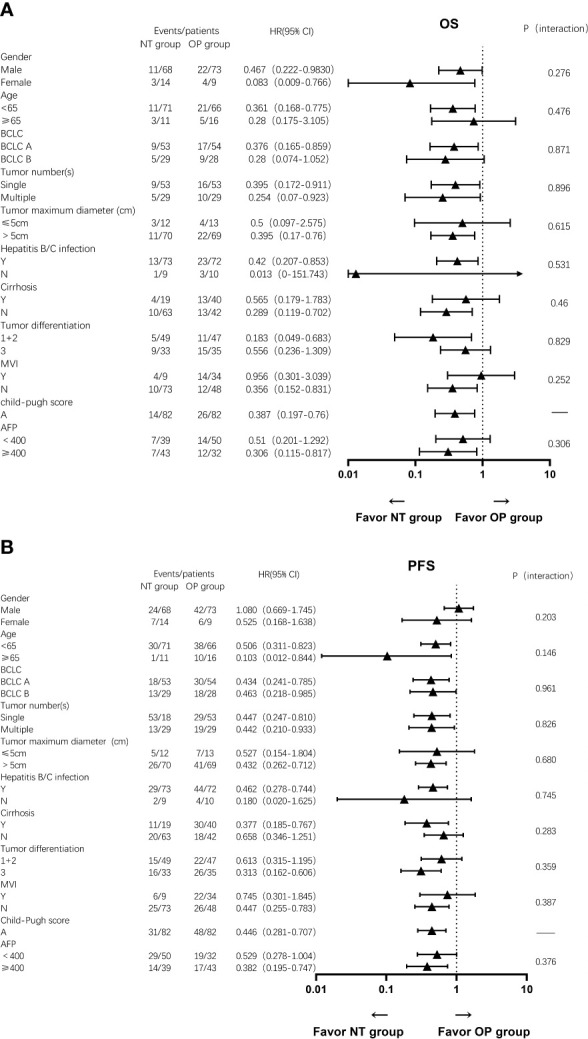
Subgroup analysis of **(A)** OS and **(B)** PFS for patients in the NT and OP groups.

There are subgroup analyses of patient outcomes in **Figure 5** (**Figure 5**). Utmost patients can have better OS and PFS benefits from the NT group. Although some accepted neoadjuvant therapy patients take the disadvantage for OS with these characters, such as age <65, patients of BCLC B stage, tumor size ≤5 cm, cirrhosis, high-level differentiation, positive MVI, and AFP <400. Similarly, gender, tumor size ≤5cm, without HBV/HCV infection, none of cirrhosis, low or middle level differentiation, positive MVI, and AFP<400 appeared to influence the advantages of neo-adjuvant therapy on PFS, but again without statistical significance.

### Safety analysis

There were no significant differences in individual AE frequencies between NT and OP groups (**Table 2**). Moreover, all AEs were mild and treated during hospitalization. In both groups, the most common AEs were pain, hyperbilirubinemia, anemia, and elevated serum liver enzymes.

**Table 2:** Summary of treatment-related adverse events.

	NT group (n = 82)						OP group (n = 82)			p-Value
	Grade 1		Grade 2	Grade 3	Grade 4	Grade 1	Grade 2	Grade 3	Grade 4	
ALT	29		4	2	0	32	8	4	1	0.588
ALB	36		0	0	0	33	0	0	0	–
Tbil	20		15	2	0	24	24	8	0	0.324
CREA	2		0	0	0	2	2	0	0	0.221
HGB	26		13	1	0	38	12	0	0	0.332
PLT	6		3	0	0	2	3	0	0	0.334
Infection	3		0	0	0	4	0	1	0	0.408
Pain	4		0	0	0	16	1	0	0	0.619
Edema	7		0	0	0	8	2	0	0	0.208
Emesis	3		1	0	0	2	0	0	0	0.439
Nausea	0		1	0	0	1	2	0	0	0.505
Neutropenia	3		0	0	0	0	0	0	0	–
Cough	4		4	0	0	7	3	0	0	0.387
Constipation	8		0	0	0	4	1	0	0	0.188
Diarrhea	3		0	0	0	7	0	0	0	–
Hemorrhage	1		1	2	0	1	0	1	1	0.525
Hypertension	1		0	1	0	2	1	1	0	0.687

## Discussion

Liver transplantation and hepatectomy are the current curative treatments for HCC but only HCC patients who meet the Milan/UCSF criteria are eligible for liver transplantation and those beyond the Milan/UCSF criteria are at higher risk of recurrence after hepatectomy (14, 15). In China, few patients receive successful liver transplantation due to a shortage of donors and high incidence of HBV, which is exclusionary according to the Milan criteria (16). Further, patients may progress beyond the Milan criteria while waiting for liver transplantation.

There are several neoadjuvant therapies that may improve outcome for HCC patients. In addition to preoperative TACE, several new potential adjuvant or first-line therapies are available for HCC, including Locally Active Agent for Tumor Treatment and Eradication (LATTE), another percutaneous locoregional therapy. Compared to TACE, LATTE is relatively simple, requiring only an ultrasound to inject chemotherapy drugs into the tumor tissue. For patients, it may enable liver transplantation or hepatectomy, decrease surgical risk, and reduce the financial burden on patients. However, additional safety and efficacy data are required (17). Immunotherapy, such as immune checkpoint inhibitors (PD-1 and PD-L1), is another potential treatment, but many patients are insensitive to single immunotherapy cycles. Therefore, combination therapy, including novel drugs, may be a more promising research area. Recently, immunotherapy based on natural killer (NK) cells has been examined for liver cancer treatment (18, 19).

Moreover, imaging examination is essential to visually observe tumor response in patients before surgery. Radiomics can transform images into high-dimensional mineable data to monitor the differentiation in the tumor tissue and adjacent tissue, providing objective criteria for the study (20). A recent research has proved that BCLB stage, serum of AFP, tumor location, and other factors are significant factors for tumor response after TACE in HCC patients, which is similar to our findings (21). Therefore, establishing a clinical-radiological model through screening clinical data combined with radiomics may predict the survival in clinical treatment (22).

Neoadjuvant treatment is mainly applied for patients with unresectable HCC, while the safety and efficacy of its application in patients with resectable HCC remains controversial (23). Neoadjuvant TACE is one of the effective treatments for patients with unresectable HCC, potentially creating opportunities for liver resection (24). For this study, we substantiated that preoperative neoadjuvant TACE in patients with resectable HCC with multifocal lesions or large isolated lesions larger than 5 cm provided better survival benefit. In addition, this study shows that neoadjuvant TACE has admissible safety record and is well tolerated.

TACE is widely acknowledged as one of the most effective local treatments for patients with unresectable HCC. However, it is controversial whether patients with resectable HCC should receive preoperative TACE preoperatively. Some studies have reported that preoperative TACE has adverse effects, such as perihepatic adhesions that make surgery more difficult, increase the risk of liver injury and liver failure, or delay surgery, thereby allowing continued tumor growth (25–27). Further, a meta-analysis concluded that HCC patients undergoing hepatectomy do not necessarily derive a survival advantage from preoperative TACE (28). Therefore, it is critical to identify those patient groups most likely to benefit from neoadjuvant TACE (29–33). A study by Guo (11) and colleagues using propensity score matching found that preoperative TACE improved RFS (p = 0.002) and OS (p = 0.003) in the patients. In our study as well, patients with resectable BCLC A stage HCC beyond the Milan criteria who received preoperative TACE achieved a significant survival advantage in OS and PFS at 1, 2, and 3 years post-treatment, although there was a decreasing trend after 3 years. The efficacy of preoperative TACE for patients with resectable HCC beyond the Milan criteria may also be related to the number of TACE sessions, as it has been suggested that more than two TACE sessions can improve the clinical outcomes of HCC patients (25, 34) In this study, patients in the NT group received at least two preoperative neoadjuvant TACE, which may also account for the better survival benefit.

We also found no statistically significant differences in AE frequency profile between NT and OP groups, indicating that hepatectomy was the main cause of postoperative complications. Similar to previous reports on hepatectomy, most of the complications were grade 1 or 2, most frequently liver dysfunction, anemia, and hypoproteinemia (35). Thus, neoadjuvant TACE is safe and well tolerated by HCC patients with resectable tumors but beyond the Milan criteria.

This study has several limitations. First, cirrhosis was less common in the NT group, which may have contributed to the improved outcome. However, subgroup analyses indicated that neither influenced the group difference in OS or PFS. Second, patients were recruited from two clinical centers in China, which may have introduced selection bias, especially from ethnicity. The efficacy and safety of preoperative neoadjuvant TACE for patients with resectable BCLC stage A/B HCC beyond the Milan criteria should be evaluated in different ethnic populations and between patients with and without cirrhosis.

In conclusion, this study suggests that preoperative neoadjuvant TACE can improve the survival rate of patients with resectable BCLC stage A/B HCC beyond the Milan criteria.

## Data availability statement

The original contributions presented in the study are included in the article/supplementary material. Further inquiries can be directed to the corresponding authors.

## Ethics statement

The studies involving human participants were reviewed and approved by the First Affiliated Hospital of Guangzhou University of Chinese Medicine. The patients/participants provided their written informed consent to participate in this study. Written informed consent was obtained from the individual(s) for the publication of any potentially identifiable images or data included in this article.

## Author contributions

Conception: CF and CZ; Financial support: CZ; Collection and assembly of data: CF, RL, KF, JW, CC, and RY; Data analysis and interpretation: CF, RL, YZ, and HS; Manuscript writing: CF, RL, JW, and SL. All authors contributed to the article and approved the submitted version.
